# Metabolic support in the critically ill: a consensus of 19

**DOI:** 10.1186/s13054-019-2597-0

**Published:** 2019-09-18

**Authors:** Jan Wernerman, Kenneth B. Christopher, Djillali Annane, Michael P. Casaer, Craig M. Coopersmith, Adam M. Deane, Elisabeth De Waele, Gunnar Elke, Carole Ichai, Constantine J. Karvellas, Stephen A. McClave, Heleen M. Oudemans-van Straaten, Olav Rooyackers, Renee D. Stapleton, Jukka Takala, Arthur R. H. van Zanten, Paul E. Wischmeyer, Jean-Charles Preiser, Jean-Louis Vincent

**Affiliations:** 1 Department of Anaesthesia and Intensive Care Medicine, Karolinska Institutet, 14186 Stockholm, Sweden; 2 Division of Renal Medicine, Brigham and Women’s Hospital, Harvard Medical School, Boston, MA USA; 3 General ICU, Hôpital Raymond Poincaré APHP, Garches, France; 4 School of Medicine Simone Veil, University Paris Saclay - UVSQ, Versailles, France; 5 Clinical Division and Laboratory of Intensive Care Medicine, Department of Cellular and Molecular Medicine, KU Leuven, 3000 Leuven, Belgium; 6 Department of Surgery and Emory Critical Care Center, Emory University School of Medicine, Atlanta, GA USA; 7 Department of Medicine and Radiology, Royal Melbourne Hospital, The University of Melbourne, Melbourne Medical School, Parkville, VIC 3050 Australia; 8 ICU Department, Nutrition Department, Universitair Ziekenhuis Brussel, Vrije Universiteit Brussel, 1090 Brussels, Belgium; 9 Department of Anaesthesiology and Intensive Care Medicine, University Medical Center Schleswig-Holstein, Campus Kiel, 24105 Kiel, Germany; 10 Department of Anesthesiology and Intensive Care Medicine, Adult Intensive Care Unit, Université Côte d’Azur, Nice, France; 11 Division of Gastroenterology and Department of Critical Care Medicine, University of Alberta Hospital, University of Alberta, Edmonton, AB Canada; 12 Division of Gastroenterology, Hepatology, and Nutrition, University of Louisville, Louisville, KY USA; 13 Department of Intensive Care, Amsterdam UMC, VU University, Amsterdam, Netherlands; 14 Anesthesiology and Intensive Care, Department of Clinical Science Intervention and Technology (CLINTEC), Karolinska Institutet, Huddinge, Sweden; 15 Division of Pulmonary and Critical Care Medicine , Department of Medicine, University of Vermont College of Medicine, Burlington, VT USA; 16 Department of Intensive Care Medicine, Inselspital, Bern University Hospital, University of Bern, CH-3010 Bern, Switzerland; 17 Department of Intensive Care Medicine, Gelderse Vallei Hospital, 6716 RP Ede, Netherlands; 18 Department of Anesthesiology and Surgery, Duke Clinical Research Institute, Duke University School of Medicine, Durham, NC USA; 19 Department of Intensive Care, Erasme Hospital, Université libre de Bruxelles, 1070 Brussels, Belgium

**Keywords:** Relevant outcomes, Metabolomics, Autophagy, Personalized care, Protein requirements, Permissive underfeeding, Gut dysfunction

## Abstract

Metabolic alterations in the critically ill have been studied for more than a century, but the heterogeneity of the critically ill patient population, the varying duration and severity of the acute phase of illness, and the many confounding factors have hindered progress in the field. These factors may explain why management of metabolic alterations and related conditions in critically ill patients has for many years been guided by recommendations based essentially on expert opinion. Over the last decade, a number of randomized controlled trials have been conducted, providing us with important population-level evidence that refutes several longstanding paradigms. However, between-patient variation means there is still substantial uncertainty when translating population-level evidence to individuals. A cornerstone of metabolic care is nutrition, for which there is a multifold of published guidelines that agree on many issues but disagree on others. Using a series of nine questions, we provide a review of the latest data in this field and a background to promote efforts to address the need for international consistency in recommendations related to the metabolic care of the critically ill patient. Our purpose is not to replace existing guidelines, but to comment on differences and add perspective.

## Introduction

During the last decade, understanding of critical illness-related metabolic changes has evolved following advances based on novel discoveries and clinical evidence from prospective randomized controlled trials (RCTs). In this review, we discuss the influence of these recent findings on the daily care of critically ill patients, focusing on nutrition as a cornerstone of metabolic care. Integrating clinical trial data into individual patient care is complex, so we have tried to group issues together into discrete, clinically relevant questions, but acknowledge that some aspects will inevitably fall into more than one category.

### Question 1: Should outcomes in clinical trials on metabolic care be more patient-centered?

Medical progress has enabled effective treatment of older and frail patients with higher severity of disease than in the past. Since the trajectory of many ICU survivors is characterized by long-term decline, survival as a clinical trial outcome is not sufficient; functional, patient-centered outcomes, such as years with good quality of life and physical/cognitive function, also need to be assessed [1, 2]. In 170 published RCTs of nutritional therapy in the critically ill, the most common primary endpoint was nutrition-related complications followed by mortality and length of stay [3]; among secondary endpoints, functional and/or long-term endpoints were uncommon.

Performing comparative effectiveness research using functional outcomes can be difficult for ICU nutrition interventions for several reasons, including that these endpoints are difficult to define objectively and baseline status is currently often not quantified. The complexity of the task to integrate results from scientific studies into patient care should not be underestimated; comparing control and intervention groups in randomized nutrition trials, as well as in meta-analyses and other cohort studies, can be especially challenging [4]. Better validated and standardized physical function outcomes specific for this patient population are needed. One key feature of critical illness is the extensive loss of muscle mass beyond that attributable to immobilization alone [5]. Loss of muscle mass portended high mortality for ICU long-stayers of the past and remains important today [6], but is no longer directly associated with mortality, rather with functional autonomy. The associated sarcopenia is a major contributor to the slow rehabilitation process [7]. The lack of functional data in most studies is thus regrettable when metabolic interventions aim to attenuate the loss of muscle mass or lean body mass. New non-invasive technologies may be helpful in monitoring muscle wasting [8]. Frailty is also insufficiently represented in the present risk scores (i.e., APACHE, SAPS), and measures of frailty need additional development and validation.

Another problematic issue in ICU nutrition studies is the time span between the nutritional intervention during the initial weeks of critical illness and outcomes recorded several months later [2]. Because ICU patients are likely to stay in the hospital at least twice as long as they stayed in the ICU, treatment during the recovery period and evaluation of patient-oriented outcomes during follow-up is crucial. It is not uncommon that the nutrition regimen given during the ICU stay is immediately discontinued after hospital or even ICU discharge and nutritional deficits may persist or even worsen [9]. A better record of the continuation of nutrition from the protocol period to the study endpoint would greatly benefit the validity of these studies.

### Question 2: Have we characterized the underlying biochemistry sufficiently?

Almost 100 years ago, David Cuthbertson and Francis Moore described a three-phase model of the metabolic response to acute illness: an early acute phase, characterized by instability, resistance to anabolic stimuli, and decreased metabolism, is followed by a catabolic phase in the ICU and post-ICU, and finally, by a recovery, anabolic phase. Many of the principles underlying this model remain true [10]. It is plausible to consider that nutrition treatment should be different in these different phases [11] or, because the duration of each of the three phases varies among individuals, as a result of different presenting illnesses or injuries (e.g., trauma patients have a higher resting metabolic rate than surgical or medical patients) as well as different patient ages and body weights [12], that it should be adjusted according to the individual metabolic profile of a patient at a certain time point. The challenge is how to detect and define these profiles in individual patients, and how to adjust the protocols of nutrition studies accordingly.

Metabolomics, the identification and quantification of metabolites within a biological system, is a promising means of characterizing patients by their metabolic profile, identifying clinical risk predictors, stratifying disease severity, increasing understanding of the mechanism of disease and examining the response to treatment or intervention [13]. Metabolomics primarily focuses on the final products of metabolism measurable at the organ level or more commonly in the blood. Metabolomic studies in sepsis have begun to characterize the biochemistry of critical illness severity and outcome [14–16]. However, issues with small sample sizes, single time points, sample handling, analytical precision, metabolite variability, large ranges of metabolite concentration, and potential false discovery with multiple hypothesis testing currently limit the applicability of this approach. Furthermore, critical illness is a collection of syndromes of differing causes with a lack of clearly defined diagnostics; this complicates metabolic characterization. More fundamentally, even the most sophisticated observational research will not be able to distinguish adaptive metabolic responses to critical illness from responses that contribute to ongoing harm. Data quality can be enhanced by collaborating with specialists in mass spectrometry and bioinformatics and including clinical parameters in the data analysis while adjusting for multiple testing for metabolites in each sample. Discovery can be enhanced by integrating biomarker data and measuring metabolite changes at multiple time points and post-intervention. Generalizability can be improved with larger sample sizes and using naïve replication cohorts. Future application of multi-omics technology (metabolomics, transcriptomics, epigenomics, genomics) to existing biorepositories and in interventional trials has the potential to boost focused studies in critical illness, which may reveal potential new mechanisms, diagnostic and therapeutic opportunities. While omics techniques in combination with bioinformatics may be able to identify possible new mechanisms or pathways that are affected and directed, mechanistic studies are needed to verify them.

### Question 3: How relevant is autophagy?

Cell metabolism during stress is altered to favor delivery of energy and essential metabolic substances to vital organs, rather than to fat and muscle, to enhance the chance of survival. The neuroendocrine response to critical illness is marked by increased gluconeogenesis, glycogenolysis, and concomitant insulin resistance, which can contribute to hyperglycemia in severe illness. Critical illness dramatically increases oxidative stress, leading to DNA damage in addition to the oxidization of lipids and proteins. A subsequent DNA damage response is choreographed to protect the cell, which is regulated by differential phosphorylation and ubiquitination. Severe critical illness-related mitochondrial dysfunction results in ATP depletion that is hypothesized to contribute to organ dysfunction [17].

**Fig. 1:**
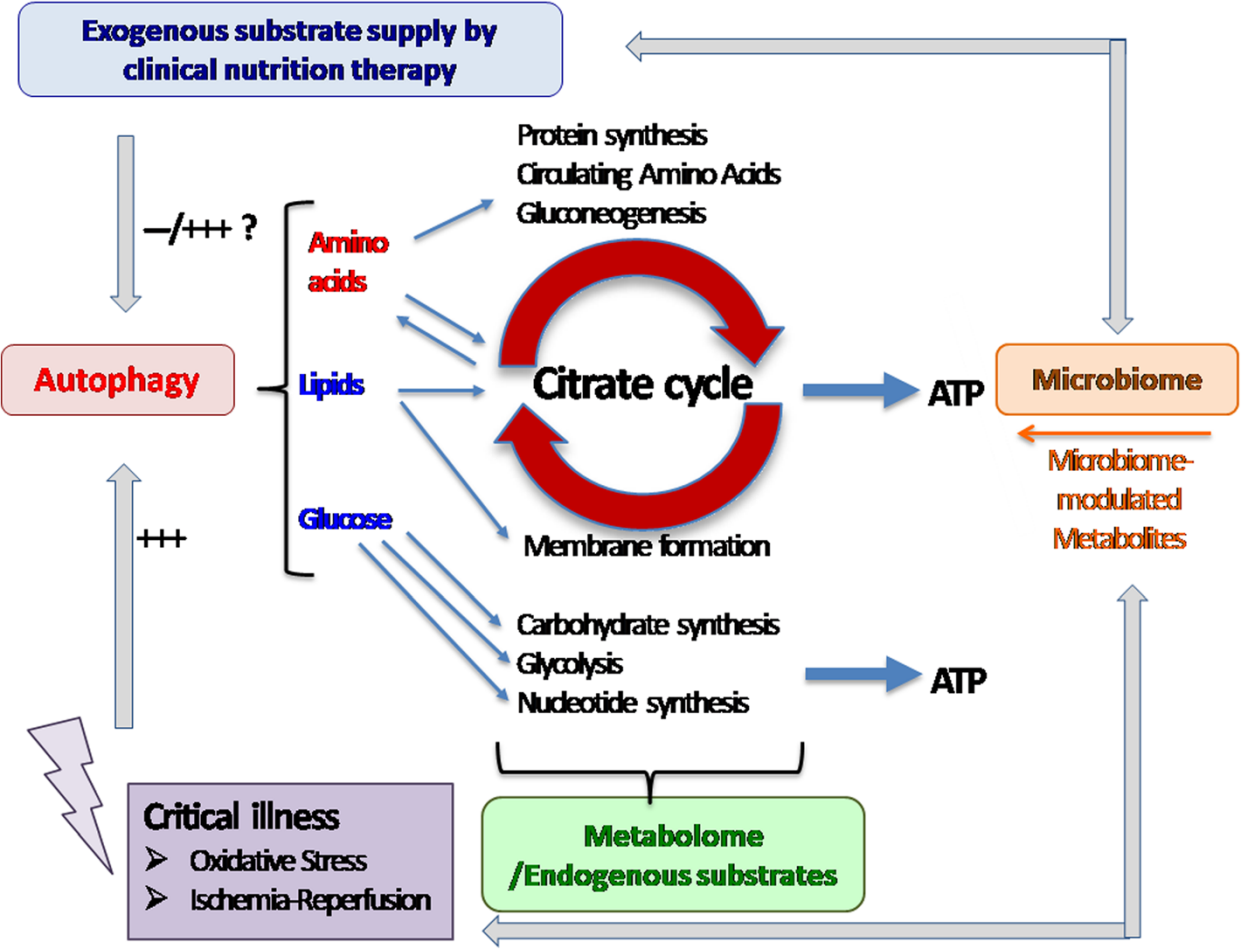
Autophagy, metabolome, microbiome—the interplay of endogenous and exogenous substrates. Critical illness triggers autophagy, which is concomitantly depressed by exogenous substrates. Intermediary metabolites can be captured in a more sophisticated approach summarized in the metabolome. Further, microbiome-modulated metabolites may influence the metabolomic pool and vice versa be influenced by the degree of underlying critical illness per se and associated treatment, particularly the use of antimicrobial agents

Autophagy is important for the maintenance of cellular and mitochondrial function [18] and is another aspect of cell metabolism that is altered during stress. The highly conserved autophagy pathway degrades cytoplasmic components and damaged organelles and recycles long-lived and damaged proteins. Autophagy is present at low levels in almost all cells and is crucial to cellular integrity and function. Critical illness is to some extent an autophagy-deficient phenotype. Autophagy is stimulated by starvation, oxidative stress, and infection and suppressed by nutrients, insulin therapy, and, most likely, other pharmacological treatments [19] (Fig. 1). The induction of autophagy by nutrient starvation leads to the provocative question of whether the feeding strategy should take into account autophagy activation and inhibition. Indeed, further suppression of autophagy with exogenous nutrients may explain why some strategies of enhanced medical nutrition—particularly amino acids—resulted in delayed recovery or harm as observed in some interventional nutrition trials in critically ill adults (EPaNIC) [20] and children (PEPaNIC) [21]. Currently, however, there is discordance between the evaluation of autophagy and the clinical relevancy of what can be measured. Moreover, suppression of autophagy is unlikely to be the only mechanism explaining the increased incidence of infections (EPaNIC & PEPaNIC) or even mortality (INTACT) [22] observed with early enhanced feeding.

### Question 4: How important is gut dysfunction?

Gut dysfunction occurs as a result of acute gastrointestinal injury in response to critical illness [23] and is often associated with impaired effective delivery of enteral nutrients. Gut dysfunction in the critically ill includes gut dysmotility, feeding intolerance, reduced small intestinal macronutrient absorption, and altered defecation resulting in constipation or diarrhea. Gut dysmotility (impaired gastric emptying, altered intestinal contractility) is common in critically ill patients and is exacerbated by intra-abdominal hypertension/compartment syndrome, positive pressure ventilation, high-dose catecholamines, intravenous narcotics, and fluid overload. Small intestine macronutrient absorption is impaired in critical illness due to abnormal luminal digestion, as a result of gut dysmotility, gut microbiome changes (see next section), and pancreatic insufficiency. Such impaired absorption may lead to malnutrition and clinical symptoms including abdominal distension, pain, and diarrhea. Moreover, mucosal factors involved in brush border enzymatic digestion, nutrient transport, and mesenteric blood flow are altered by small intestinal nutrient absorption. Carbohydrate malabsorption in the ICU is characterized by a reduction in the number of small intestinal glucose transporters [24]. Amino acid and fat absorption are also attenuated [25].

The impact of duodenal or jejunal feeding on the interaction between nutrients and gastric secretion and the absorption of macronutrients and vitamins has not been widely studied, and the use of post-pyloric feeding to decrease the rate of pneumonia is controversial [26]. Use of prokinetic agents (erythromycin, metoclopramide) significantly reduces feeding intolerance and risk of high gastric residual volumes [27]. However, intolerance to enteral feeding, particularly early in critical illness, may be considered an adaptive response, which may challenge the classical dogma of full early enteral nutrition in the acute phase. One key problem with conducting clinical trials in this field is that gut dysfunction is difficult to evaluate objectively. This was noted by the developers of the sequential organ failure assessment (SOFA) who despite considering gut dysfunction as “very important” chose not to include it in the score [28]. Intestinal-type fatty acid-binding protein (I-FABP) may be a potential marker of enterocyte damage [29].

### Question 5: How important is the gut microbiome in acutely ill patients?

The gut microbiome has an increasingly recognized role in maintaining homeostasis [30]. Cross-talk signaling between commensal organisms and pattern recognition receptors on the gut epithelium and at the epigenetic level sustains all aspects of mucosal defense, symbiosis, and appropriate immune responses. This intimate cross-talk may be further driven by microbiota-mediated metabolite secretion and signaling, profoundly influencing (patho)physiology, including endocrine, metabolic, and nervous system function as a cause as well as a consequence of critical illness (Fig. 1). Alterations in microbiome-associated metabolite levels and activity are implicated in the pathogenesis of a growing number of illnesses. In critical illness, the microbiome undergoes a loss of diversity, termed dysbiosis, loss of site specificity, loss of key potentially beneficial commensal organisms, and a shift toward dominant pathogens (“pathobiomer”) [31]. Such alterations are associated with adverse outcomes, and distinct patterns of microbial diversity may serve as personalized biomarkers of prognostic value.

Enteral nutrition holds promise to preserve microbiome diversity during critical illness by shifting away from stress conditions of luminal nutrient scarcity and may positively influence microbiome-mediated metabolite production. Probiotics may also improve microbiome restoration and outcomes, as shown in recent meta-analysis data [32]. Limitations of these data include lack of a consistent probiotic strain or dose across most conducted trials, and further, adequately powered trials are needed. Fecal microbiota transplantation may be a novel therapeutic strategy in these patients via alteration of microbial ecology and restoration of the microbiome diversity. Although fecal transplantation activates immune clearance of pathogens, restores crypt commensal microbiota, establishes stem cell regenerative capacity, and suppresses inflammation, the literature in critically ill patients is scant. There is also potential for harm when the gut barrier fails, such that fecal transplantation should not currently be performed outside of a well-conducted study or trial [33].

### Question 6: What do we mean by individualized management?

**Fig. 2:**
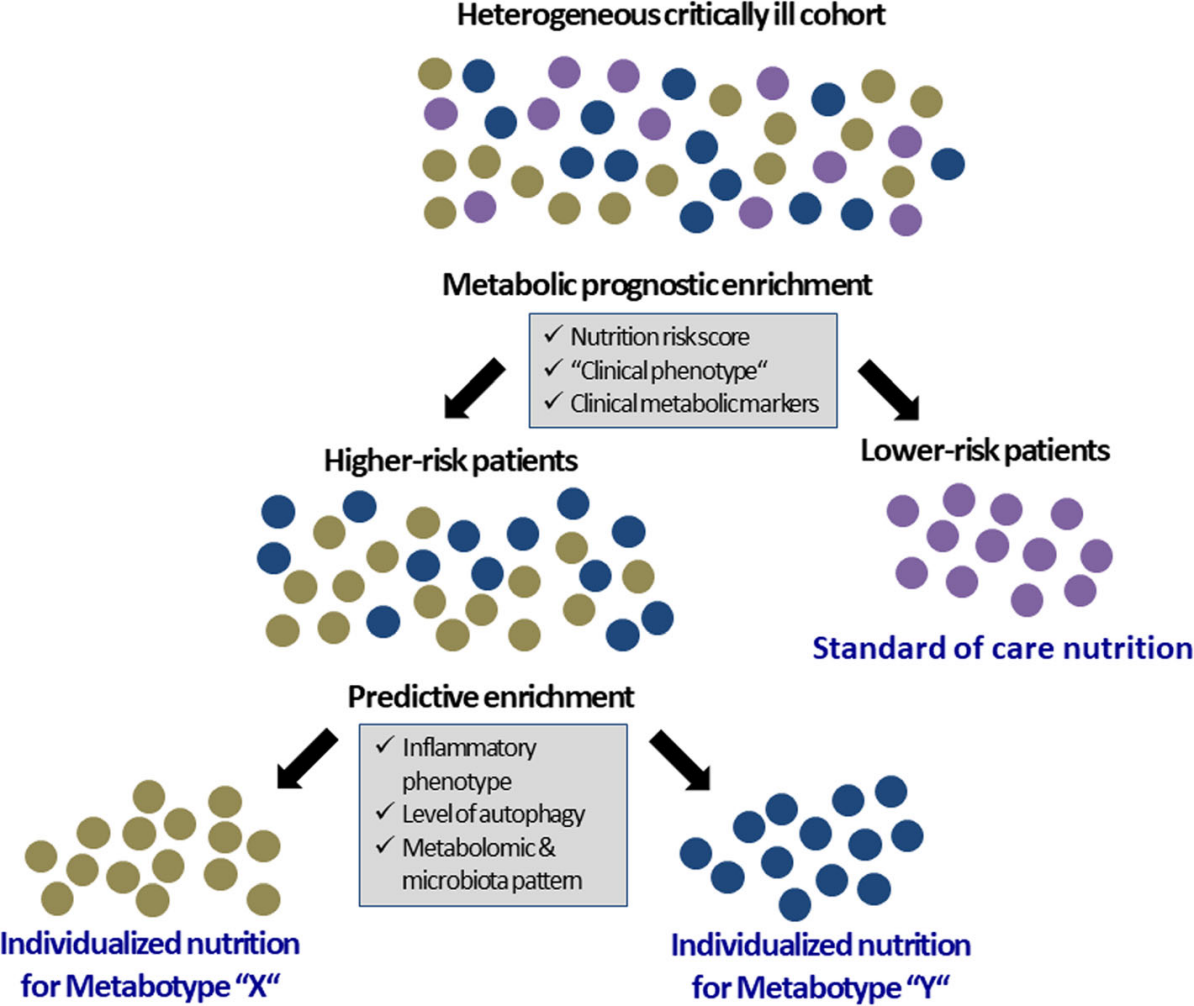
Proposed future approach to the application of prognostic and predictive enrichment strategies for metabolic care and individualized nutrition in critical illness. Individuals are represented by circles filled with different colors to reflect patients with similar metabolic prognostic and predictive characteristics

Provision of adequate nutrition has an important role to play in the move toward individualized treatment and development of precision intensive care medicine (Fig. 2). However, to be able to implement this, we need tools to monitor individual patient needs, their potential to utilize the given nutrition, and their tolerance to feeding. Yet, only a handful of biochemical markers of metabolic function and nutrition, most notably glucose, triglycerides, urea, lactate, electrolytes (PO_4_, Mg, K), and oxygen utilization (PaCo_2_, VO_2_, and VCO_2_), in addition to exogenous insulin demand and measurement of energy expenditure, are available at the bedside. No tools for assessment of more specific aspects of metabolism and nutrition, such as protein needs, are available. Energy expenditure may be measured in the individual patient using indirect calorimetry [34]. Modern devices are highly accurate and user friendly, whereas the large numbers of suggested equations to estimate energy expenditure are less reliable [35]. However, although indirect calorimetry is easy to perform in mechanically ventilated patients at moderate FiO_2_, it is more challenging in spontaneously breathing patients requiring the use of masks, mouthpieces or hoods, where the use of oxygen supplementation represents a problem. Nevertheless, indirect calorimetry is the only tool available to measure the actual energy expenditure of a patient in everyday clinical practice in order to tailor nutrition therapy. Some studies using indirect calorimetry have suggested reductions in infectious complications and cost savings with individualized nutrition [36–38]; however, this type of individualized feeding has so far not been tested in adequately powered RCTs [39, 40].

Caution is necessary when transforming the measured level of energy expenditure into a caloric target for nutrition therapy. During critical illness, exogenous nutrients do not suppress endogenous substrate mobilization, which is closely related to insulin resistance in critical illness [41]. This phenomenon may lead to “overfeeding” if the delivered nutritional energy is equivalent to the measured energy expenditure; therefore, it is rational to monitor the patient’s metabolic tolerance in order to avoid overfeeding or refeeding particularly in the early acute phase. Unfortunately, there is no good marker at present of the amount of endogenous substrate mobilized or for how long this particular alteration in metabolism persists. Refeeding hypophosphatemia has been shown to be associated with unfavorable outcomes when the caloric intake exceeds 50% of the caloric target in the acute phase even when hypophosphatemia is corrected [42–44]. Another concern is the U-shaped curve describing the relationship between caloric intake and outcomes in observational studies [45, 46]. It is relevant to note that observational data suggest that administration of > 80% of energy expenditure is associated with higher mortality, although a recent large RCT did not reveal any mortality difference between 22 and 30 kcal/kg/24 h [47].

### Question 7: Is permissive underfeeding relevant?

Hypocaloric or permissive underfeeding is defined as providing < 40–60% of the calories required for daily energy expenditure. Existing RCTs show no effect of permissive underfeeding on mortality, with inconsistent effects on functional outcomes, but also no harm [48, 49]. Trickle or trophic feeding is defined as providing 20 kcal/h up to 500 kcal/day via the enteral route. There may be benefits of using trophic or minimal enteral feeding, including intestinal epithelium preservation, amplified brush border enzyme secretion, augmented immune function, preservation of epithelial tight cell junctions, and limited bacterial translocation. However, recent large RCTs in the field do not favor enteral over parenteral nutrition (CALORIES, NUTRIREA-2) or high over moderate caloric intake (TARGET) in terms of mortality endpoints [47, 50, 51]. Notably, none of these studies measured energy expenditure to establish caloric targets.

As the prevalence of obesity increases around the world, better understanding of appropriate nutrition therapy in obese critically ill patients is crucial [52]. Obese patients appear to be different to lean patients, with many studies repeatedly suggesting better survival, although the reasons for this “obesity paradox” are not well understood [53]. Adjustment for nutrition status abrogates the improved survival association seen in obese patients, and obese patients with malnutrition do not have a survival advantage [53]. Existing literature regarding the proposed strategy of hypocaloric (permissive), high-protein feeding in obese patients is not well substantiated by methodologically sound and adequately powered trials.

### Question 8: How can we define protein requirements?

Observational studies suggest that the daily protein intake recommended for healthy individuals is insufficient for critically ill patients [45, 46]. As a result, all existing guidelines recommend a higher protein intake in critically ill patients, varying from 1.2–2.0 g/kg/day as compared to 0.80 g/kg/day for the healthy, although these recommendations are based on weak evidence. When 24-h nitrogen balances are used as an endpoint, recommendations of > 2.0 g/kg/day of protein have been proposed [54]. Yet, in prospective randomized trials, protein doses > 1.2 g/kg/day did not provide better 7-day nitrogen balance and did not improve patient-centered outcomes [40, 55, 56].

In studies using observational registries and in selected cohorts, high-protein intake has been associated with improved survival [57]. Retrospective classification of malnutrition or nutritional risk has generated hypotheses that a high-protein intake may be particularly advantageous in these cohorts. However, several small RCTs did not demonstrate benefit with high protein [40, 58]. In contrast, there are observational studies and post hoc analyses of other RCTs suggesting that a high-protein intake, particularly in the acute phase of critical illness, may be harmful [59, 60]. This is the area where the existing guidelines and expert opinions diverge the most.

We urgently need more insight into the kinetics of protein synthesis and breakdown in relation to the phase of critical illness and dose of amino acids/protein administered. As indicated earlier, nitrogen balance may not be the ideal proxy to titrate dosing in amount or in temporal pattern. Measurements of whole-body net protein balance employing isotope-labeled amino acids may provide clarity in this situation [61, 62]. Amino acid oxidation can be measured and then repeated to form a temporal pattern. To correctly estimate the contribution of the intake to the central pool of amino acids, it is important to measure the actual enteral uptake of amino acids from the gut when enteral nutrition is given [25, 63]. Ignoring the central pool of amino acids in calculations of protein kinetics may significantly confound the results. Smaller studies using this technology will enable us to define the potential of the critically ill patient to utilize nutritional protein and amino acids. Such studies may then contribute to the design of more “successful” large interventional nutrition trials thanks to protocols more adapted to human physiology in critical illness. The effects of protein intake combined with physical activity in the acute phase as well as during rehabilitation also need to be further investigated [64, 65].

### Question 9: What about specific nutrients?

The individualized approach to nutrition also applies to micronutrients, in particular those that cannot be economized and reutilized. Supplementation of micronutrients to ensure adequate daily dietary intake (as recommended for healthy subjects [classical strategy]) must be differentiated from the effects of providing micronutrients in higher doses (pharmacological approach). The latter approach has been extensively investigated, with large RCTs not showing beneficial outcomes of pharmacotherapy [66, 67]. Use of immunomodulating micronutrients, such as glutamine and fish oils, to alter morbidity and mortality is controversial [68, 69]. However, variations in doses, timing, duration, single or cocktail use of micronutrients, and target populations of critically ill patients were noted in the studies conducted and the topic is still of great interest.

For several nutrients, low concentrations are known to be associated with unfavorable outcomes, but it is often not clear whether this is related to the deficiency or the nutrient level is a biomarker of severity [70, 71]. For example, vitamin C cannot be synthesized in the human body and consumption is increased in critical illness. Enteral uptake is limited, recycling is impaired, and vitamin C plasma concentration determinations are rarely available [72]. Single-center studies have reported dramatic benefits with pharmacological doses of vitamin C, which demand confirmation [73]. Well-designed ongoing micronutrient trials of vitamin C, vitamin D, and selenium [NCT02106975, NTC03333278, NCT03096314, NCT03188796, NCT02002247] will further our understanding of specific-nutrient delivery and potentially alter our approach to critical illness patient nutrition.

There are also endogenous substrates that may have therapeutic implications and need to be considered in special situations. In starvation, ketones may provide 50% of energy and up to 70% for the brain [74]. There are reports of beneficial effects of oral ketone supplementation in neurological conditions [75] or of intravenous ketone solutions in animal models [76]. Presently, there are no commercially available intravenous ketone preparations.

Blood lactate levels are considered a robust biomarker of altered oxygen utilization, mainly reflecting an impaired microcirculation with a local oxygen deficit. However, there are several other explanations for hyperlactatemia, such as increased aerobic glycolysis, effects of catecholamines and decreased clearance usually as a result of impaired liver function. Measuring pyruvate concentrations to determine the lactate/pyruvate ratio could help to differentiate the source of lactate, but reliable pyruvate analyses are not available. Both endogenous and exogenous lactate can be a preferential source of energy in critical metabolic situations for several organs (brain, heart, muscle). Following sodium lactate infusion, improvements in cardiac output and oxygenation occur during cardiac surgery and in patients with acute heart failure [77, 78]. Interestingly, the contribution of lactate to brain metabolism can reach up to 60% [79]. Hypertonic lactate therapy enables preferential utilization of lactate as suggested by the reduction in both brain glutamate and intracranial pressure (ICP), coupled with increased extracellular pyruvate and glucose in patients with traumatic brain injury [80]. Exogenous lactate can also be used to control ICP in acute brain injury, but the clinical implications remain to be established [81].

## Conclusion: How we see the future

**Fig. 3:**
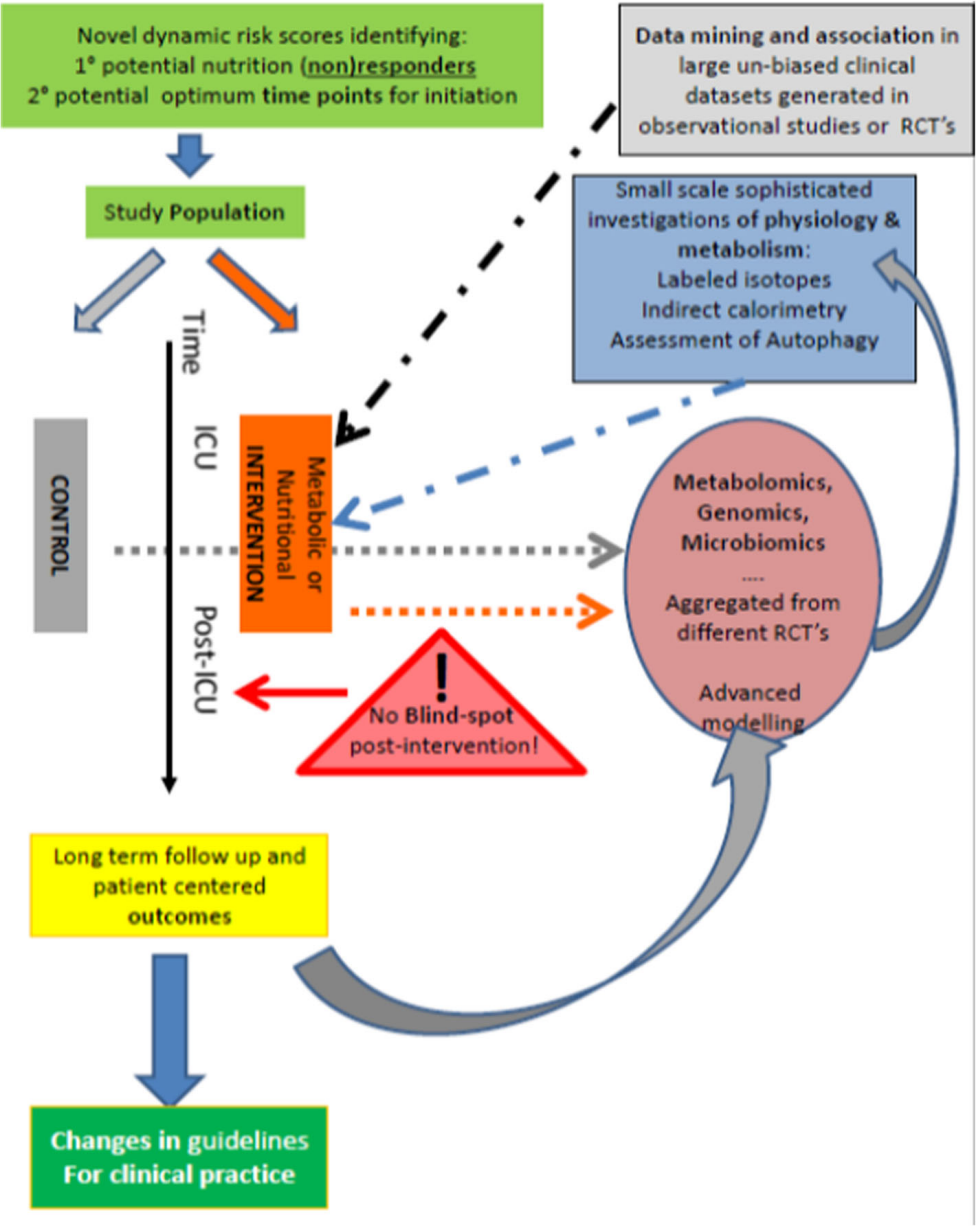
Proposed schema for the determination of optimal nutrient administration in critical illness. Approach combines metabolic or nutritional intervention with longer-term outcomes, data mining, omics, and evaluation of physiology and metabolism

Recent basic and clinical science studies have highlighted the importance of nutrition and metabolism in critical illness and the progress that is being made in our understanding of the physiology of metabolism and nutrition handling. There are more high impact clinical trials and well-designed observational studies in this field than ever before. However, despite our advances in knowledge, study results have yet to substantially alter metabolic and nutritional practice in critical illness and controversies remain. Individualizing nutrition to the patient’s specific metabolic profile and type of disease, comorbidity, and body composition seems an important future step, although will require a complete change in the way in which patients are currently managed (Fig. 3).

Multiple guidelines for nutrition in the critically ill exist, many produced and endorsed by societies in nutrition and/or critical care [11, 82–84]. However, because of the weakness of the supporting evidence and the sometimes limited clinical plausibility between nutritional interventions and the reported outcomes, experts disagree regarding optimal nutritional approaches and conflicting recommendations have sometimes been published, limiting global acceptance and application. Much as the original *Surviving Sepsis Campaign* guidelines provided general guidance for sepsis management [85] and have changed clinical practice, we, as a nutrition and metabolism community within critical care, propose to build on the areas of consensus presented here to find topics of broad agreement within the global field of metabolic support, which can serve as fundamental principles to guide practicing intensivists, enabling clinicians and researchers to clearly distinguish areas of growing certainty from those requiring further investigation.

## Data Availability

Not applicable.

## Abbreviations

ICP: Intracranial pressure
ICU: Intensive care unit
RCT: Randomized controlled trial
